# Increasing Analytical Quality by Designing a Thin-Layer Chromatography Scanner Method for the Determination of the Radiochemical Purity of Radiopharmaceutical Sodium Iodide ^131^I Oral Solution

**DOI:** 10.3390/molecules29081883

**Published:** 2024-04-20

**Authors:** Miguel Vasquez-Huaman, Américo Castro-Luna, Norma Julia Ramos-Cevallos, Donald Ramos-Perfecto, Mario Alcarraz-Curi, Jacqueline Segura-Vasquez, Danny Cáceres-Antaurco

**Affiliations:** 1 Research Institute for Pharmaceutical Sciences and Natural Resources, Faculty of Pharmacy and Biochemistry, Universidad Nacional Mayor de San Marcos, Jr. Puno 1002, Lima 15001, Peru; nramosc@unmsm.edu.pe (N.J.R.-C.); jacqueline.segura@unmsm.edu.pe (J.S.-V.); danny.caceres1@unmsm.edu.pe (D.C.-A.); 2 Faculty of Odontology, Universidad Nacional Mayor de San Marcos, Av Germán Amézaga 375, Lima 15081, Peru; dramosp@unmsm.edu.pe; 3 Faculty of Biological Sciences, Universidad Nacional Mayor de San Marcos, Av. Venezuela Cuadra 34 s/n Cercado de Lima, Lima 15081, Peru; malcarrazc@unmsm.edu.pe

**Keywords:** analytical quality by desing (AQbD), critical analytical attributes (CAA), method operable design region (MODR), radiochemical purity, thin-layer chromatography scanner (TLC-scanner), radiopharmaceutical, sodium iodide

## Abstract

The goal of this study was to apply the principles of analytical quality by design (AQbD) to the analytical method for determining the radiochemical purity (PQR) of the radiopharmaceutical sodium iodide ^131^I oral solution, utilizing thin-layer chromatography (TLC) with a radio–TLC scanner, which also enables the evaluation of product quality. For AQbD, the analytical target profile (ATP), critical quality attributes (CQA), risk management, and the method operable design region (MODR) were defined through response surface methodology to optimize the method using MINITAB^®^ 19 software. This study encompassed the establishment of a control strategy and the validation of the method, including the assessment of selectivity, linearity, precision, robustness, detection limit, quantification limit, range, and the stability of the sample solution. Under the experimental conditions, the method parameters of the TLC scanner were experimentally demonstrated and optimized with an injection volume of 3 µL, a radioactive concentration of 10 mCi/mL, and a carrier volume of 40 µL. Statistical analysis confirmed the method’s selectivity for the ^131^I iodide band Rf of 0.8, a radiochemical impurity IO_3_^−^ Rf of 0.6, a linearity from 6.0 to 22.0 mCi/mL, and an intermediate precision with a global relative standard deviation (RSD) of 0.624%. The method also exhibited robustness, with a global RSD of 0.101%, a detection limit of 0.09 mCi/mL, and a quantification limit of 0.53 Ci/mL, meeting the prescribed range and displaying stability over time (at 0, 2, and 20 h) with a global RSD of 0.362%, resulting in consistent outcomes. The development of a method based on AQbD facilitated the creation of a design space and an operational space, with comprehensive knowledge of the method’s characteristics and limitations. Additionally, throughout all operations, compliance with the acceptance criteria was verified. The method’s validity was confirmed under the established conditions, making it suitable for use in the manufacturing process of sodium iodide ^131^I and application in nuclear medicine services.

## 1. Introduction

Over the past decade, the pharmaceutical industry has placed significant emphasis on product quality, safety, and efficacy. The enhancement of product quality has been achieved through the implementation of scientific methodologies such as quality by design (QbD) [1]. The concept of QbD has been introduced to enhance both manufacturing processes and product quality. QbD concepts are defined in ICH Q8 (R1), Q9 and Q10. In late 2013 and early 2014, there was a heightened emphasis on implementing the existing QbD framework in the context of method development and analysis. Several researchers report that similar opportunities exist to apply QbD to the analysis of analytical methods as they are applied to manufacturing processes [2]. ICH Q8 (R1) defines QbD as a system of thematic approach to development that commences with well-defined objectives and emphasizes product and process understanding and process control, based on sound science and quality risk management. Similar ideologies inherent in QbD are applied in the development of analytical methods that are defined in analytical quality by design (AQbD). The AQbD process is an important stage of the quality system control strategy, although good manufacturing practices have been in place for a long time, many companies continue to face challenges related to quality control. The quality assurance professionals regard AQbD as a key solution to prevent occurrences of out-of-specifications (OOS) or out-of-trend (OOT) results, thereby minimizing the risk of method failure. The traditional approach within the pharmaceutical industry was quality by testing (QbT); however, this is gradually becoming outdated in light of the QbD philosophy, which ensures product quality but also contributes to cost reduction and time savings. Analytical techniques play a central role in the control strategy, making the adoption of AQbD crucial to enhance the concept of accurate analysis, an important aspect in the pharmaceutical development cycle [3].

Radiopharmaceuticals are medicinal agents marked with radioisotopes or radioactive compounds used in the diagnosis and/or treatment of diseases with their application conducted within nuclear medicine services. Iodine radioisotopes are widely used in nuclear medicine. In our country, ^131^I is used in diagnostic studies due to its cost-effectiveness; moreover, it is the simplest product from a reactor and has a long half-life. Sodium iodide ^131^I is an orally administered solution containing radioactive iodine, processed in the form of sodium iodide from uranium tellurium fission products. It exhibits partial uptake and iodine concentration in the thyroid gland, quantifying the iodine concentration within the gland to assess whether its function is within the normal range, elevated as in hyperthyroidism, or reduced as in hypothyroidism. Its gamma emission allows for its use in diagnostic studies. Quality control ensures that circulation or sale is not allowed until its quality has been tested and has been established as satisfactory. The types of assays include physical–chemical, biological, and nuclear controls. Among the physical–chemical controls, radiochemical purity, as described in USP43, is defined as the fraction of total radioactivity in the desired chemical form present within the radiopharmaceutical. Impurities may arise from radiopharmaceutical decomposition, solvent action, temperature, light exposure, radiolysis, or impurity labeling with the same radionuclide. The most frequently employed analytical techniques for detecting and determining radiochemical impurities in a radiopharmaceutical include precipitation, paper chromatography, thin-layer chromatography, gel chromatography, paper electrophoresis, thin-layer electrophoresis, high-performance liquid chromatography, solvent extraction, solid-phase extraction, and distillation [4,5,6,7]. For assessing the radiochemical purity employing small-sized paper and chromatographic strips, there are straightforward techniques that facilitate rapid chromatography [8]. Therefore, alternative methods or procedures can be employed to gain advantages in terms of accuracy, sensitivity, precision, selectivity, or adaptability to automation, thereby reducing computerized data. However, these alternative methods and procedures must undergo validation, in accordance with the guidelines outlined in the general chapter on the validation of pharmacopeial procedures <1225>. It must be conclusively demonstrated that they provide equivalent or superior outcomes. Consequently, the proposed method requires analytical validation. The radio–TLC scanner represents a relatively simple technique but can be highly advantageous when properly validated. Compared to other methods, it offers the significant advantage of detecting all applied radioactivity without concerns related to recovery [9].

The AQbD approach constitutes an essential step in the development and validation of routine analytical techniques. The implementation of the AQbD concept is justifiable, due to the substantial impact of numerous variables on the method’s outcomes [10]. AQbD encompasses several steps, commencing with the definition of the analytical target profile (ATP), the determination of critical quality attributes (CQAs), risk assessment, the identification of design of experiments (DoE), the optimization through the methodology response surface (MSR), AQbD method validation, and continuous improvement [11,12]. The AQbD method validation approach for radiochemical purity uses knowledge of DoE and MODR to design validation according to ICH Q2 allows. Employing this approach enables the development of an alternative analytical method that is cost-effective, swift, and straightforward, while thoroughly evaluating the analytical procedure’s performance characteristics [13].

This study focuses on the implementation of AQbD in the analytical methodology for assessing the radiochemical purity of the radiopharmaceutical Sodium Iodide ^131^I in an oral solution, using a radio–TLC scanner system, which is a versatile scanner for a reliable detection of radioisotopes on narrow strips and plates. The system serves as an ideal tool for routine quality control of ^131^I, offering a motorized NaI/PMT detector equipped with an adjustable collimator and TLC plate holders, thereby enhancing the quality control process for the radiopharmaceutical, which is intended for application in nuclear medicine.

## 2. Results and Discussion

### 2.1. Analytical Target Profile (ATP)

The identification of the ATP includes the selection of the method requirements (Table 1).

### 2.2. Critical Quality Attributes (CQA)

The CQAs of the radiochemical purity by TLC scanner method for the radiopharmaceutical sodium iodide ^131^I oral solution includes attributes and method parameters (Table 2).

### 2.3. Risk Management (QRM)

**Figure 1:**
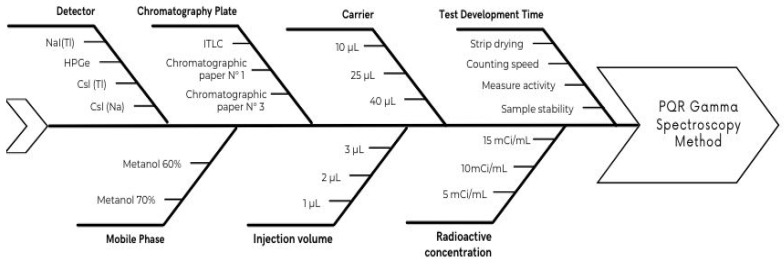
Ishikawa plot for the PQR method by gamma spectroscopy.

The AQbD approach for the TLC scanner PQR method for the radiopharmaceutical sodium iodide ^131^I oral solution includes risk identification and assessment. The aim is to comprehend and quantify the risk, for which a risk assessment tool is employed, such as the Ishikawa diagram, failure mode effects analysis, risk matrix, or similar tools, in accordance with ICH Q9 (R1) and Q8 (R2) (Figure 1) (Table 3).

### 2.4. Design of Experiments (DOE)

**Figure 2:**
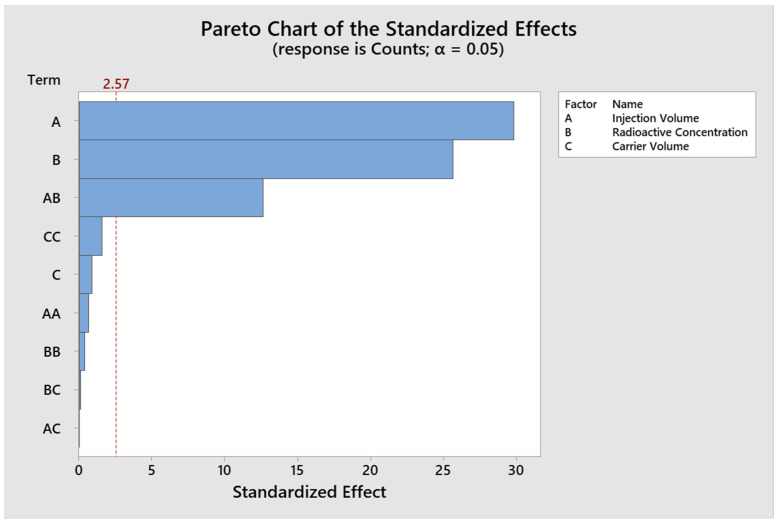
Pareto plot of significant effects for counts.

**Figure 3:**
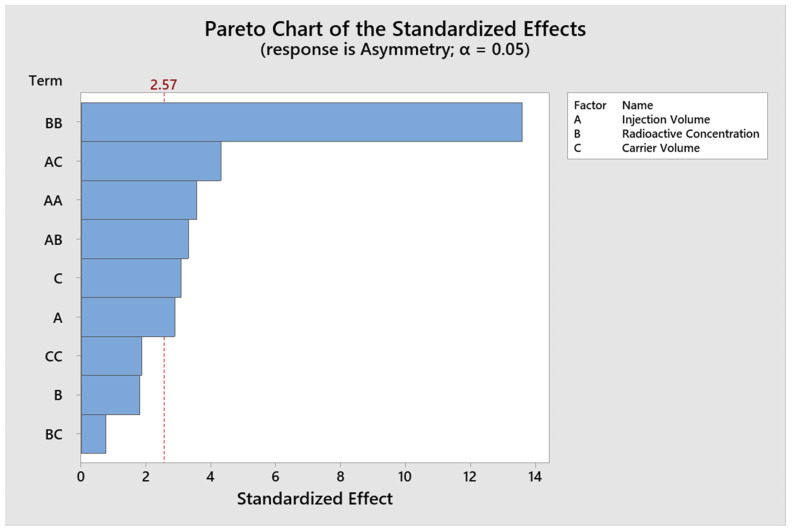
Pareto plot of significant effects for Asymmetry.

The analysis of the design of experiments using the Box–Behnken response surface (BBD) was performed using three continuous critical factors and fifteen experimental runs with the statistical program MINITAB^®^, with a random order for the response variables accounts and asymmetry. (Table 4). The results for the Response Surface Regression for the variable Counts, the significant Factors with values of *p* < 0.05 are Injection Volume and Radioactive Concentration (Figure 2). An R^2^ value of 99.71% and adjR^2^ of 99.18% were obtained. The results for the response surface regression for the response variable asymmetry, the significant factors with *p* < 0.05 values are injection volume and carrier volume (Figure 3). A R^2^ value of 97.98% and adjR^2^ of 94.36% were obtained.

**Table 4:** Design factors.

Factor	Name	Low	High
A	Injection volume	1	3
B	Radioactive concentration	5	15
C	Carrier volume	10	40

Counts

Response surface regression:

Model summary
**S****R-Quad.****R-Quad.** **(Adjusted)****R-Quad.** **(Pred)**2229.9999.71%99.18%96.19%

Asymmetry

Response surface regression:

Model Summary
**S****R-Quad.****R-Quad.** **(Adjusted)****R-Quad.** **(Pred)**0.017639297.98%94.36%70.19%

**Figure 4:**
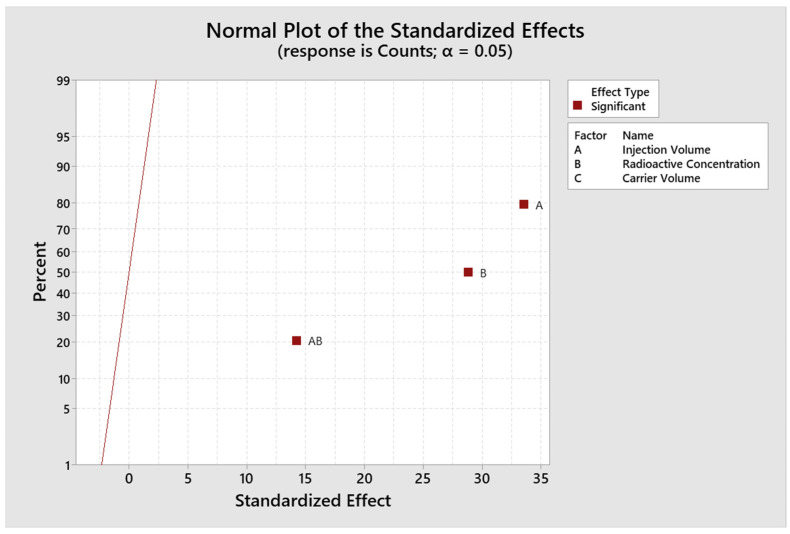
Fitted plot for standardized effects for accounts.

**Figure 5:**
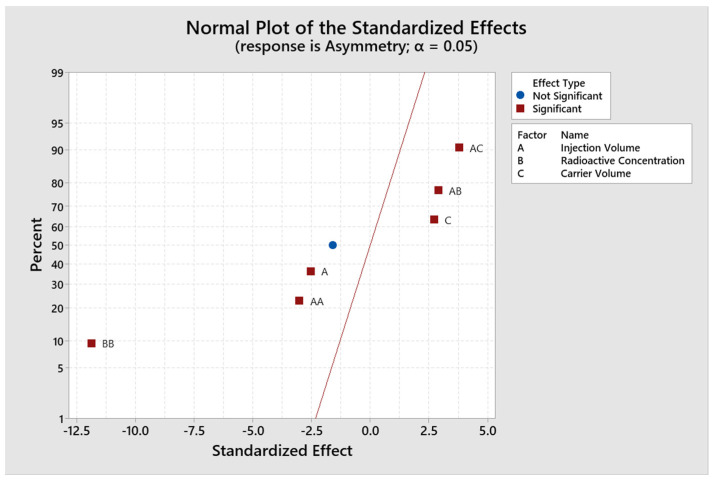
Adjusted plot for standardized effects for asymmetry.

**Figure 6:**
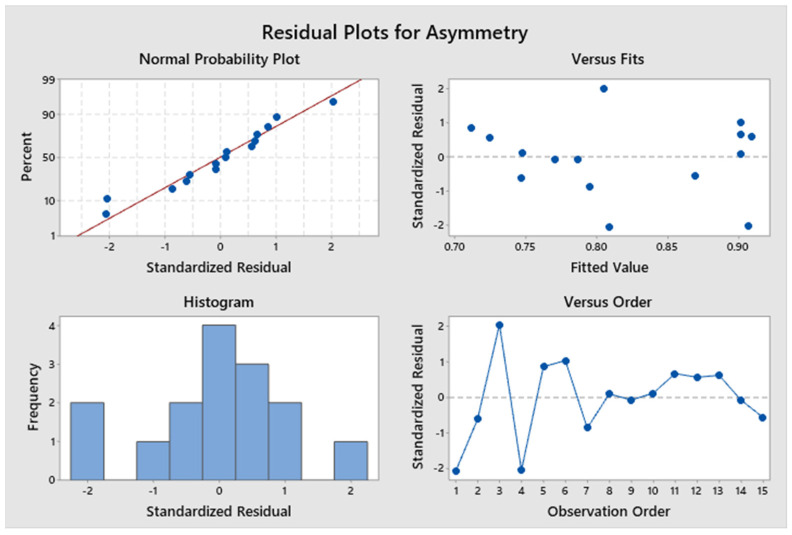
Residual plot for asymmetry.

The model is evaluated and adjusted only with those significant factors and interactions to adjust the design and recalculate the R^2^ value. The response surface regression for the response variable counts gives an R^2^ value of 99.49% and adjR^2^ of 99.35% (Figure 4), and for the response variable asymmetry gives an R^2^ of 96.35% and adjR^2^ of 92.70% (Figure 5). The residuals fit to a normal distribution; therefore, the regression performed is compliant (Figure 6).

Counts

Response surface regression:

Model Summary
**S****R-Quad.****R-Quad.** **(Adjusted)****R-Quad.** **(Pred)**1984.4999.49%99.35%98.91%

Asymmetry

Response surface regression:

Model Summary
**S****R-Quad.****R-Quad.** **(Adjusted)****R-Quad.** **(Pred)**0.020062096.35%92.70%76.07%

**Figure 7:**
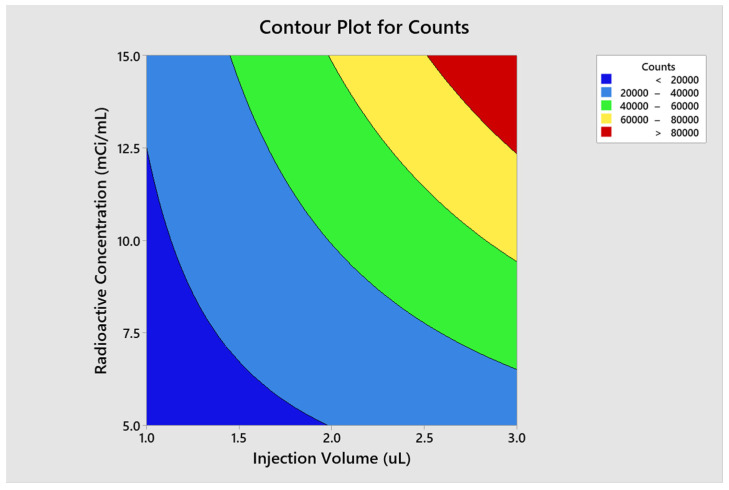
Contour plot for counts.

**Figure 8:**
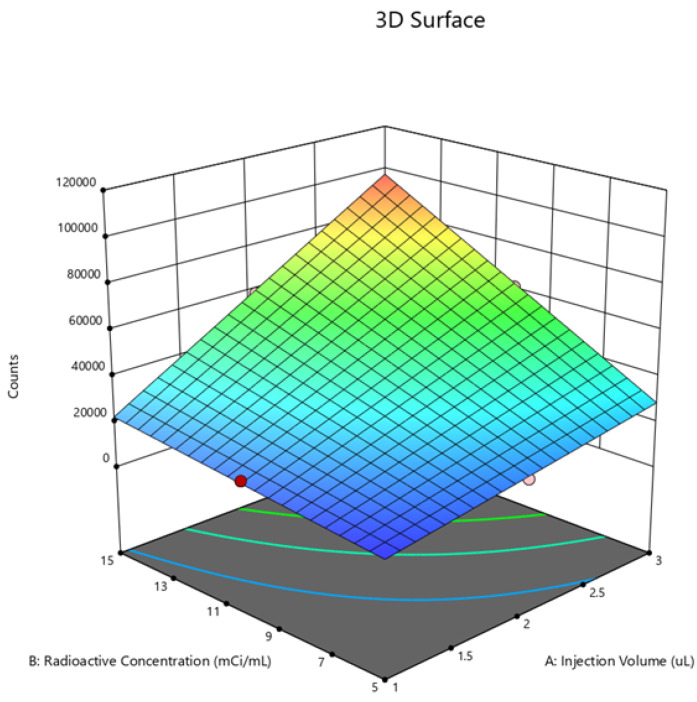
Surface chart for counts.

**Figure 9:**
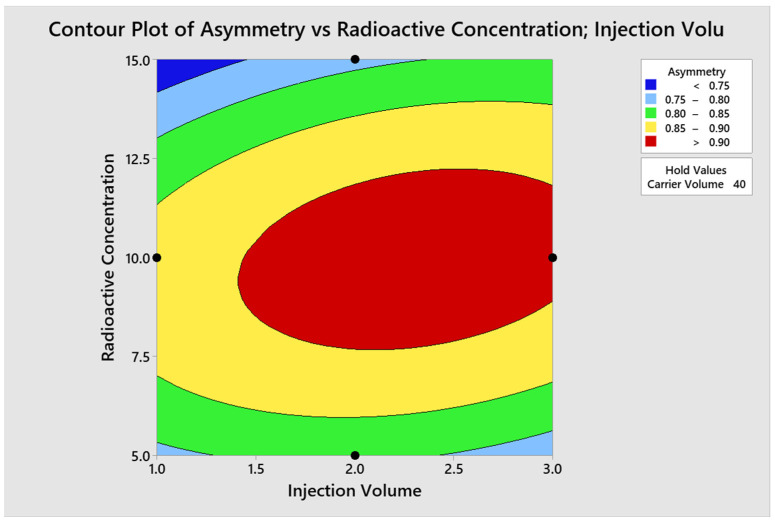
Contour plot for asymmetry.

**Figure 10:**
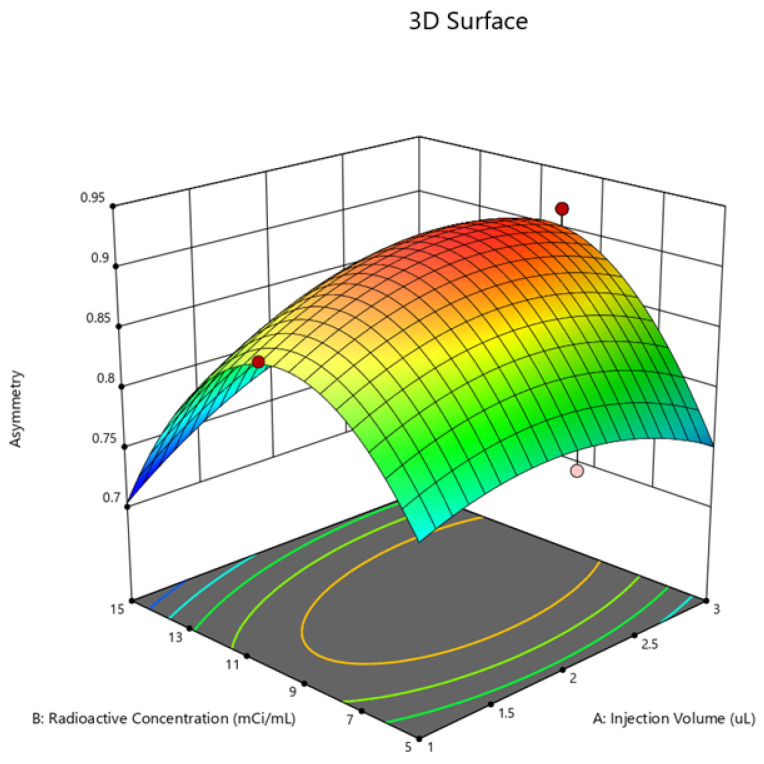
Surface plot for asymmetry.

The optimal conditions for achieving the optimal region of operation were determined through contour and surface plots for the response variables counts (Figure 7 and Figure 8) and asymmetry (Figure 9 and Figure 10).

Counts

Optimal conditions:

Asymmetry

Optimal conditions:

### 2.5. Determination of Method Operable Design Region (MODR)

**Figure 11:**
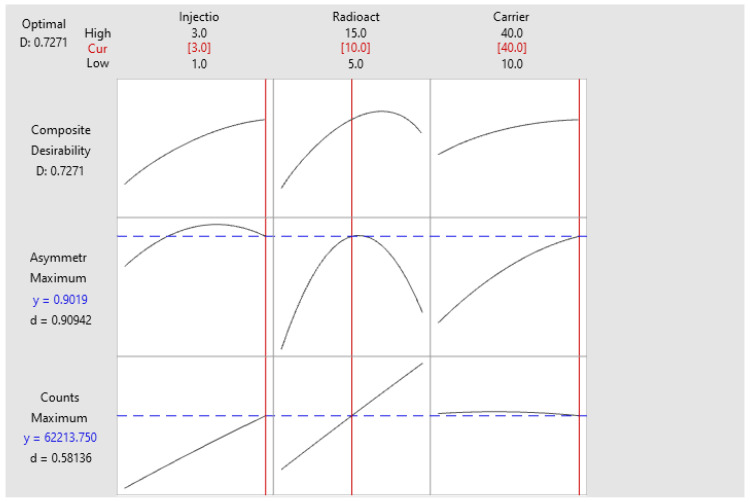
Response optimizer ^131^I.

**Figure 12:**
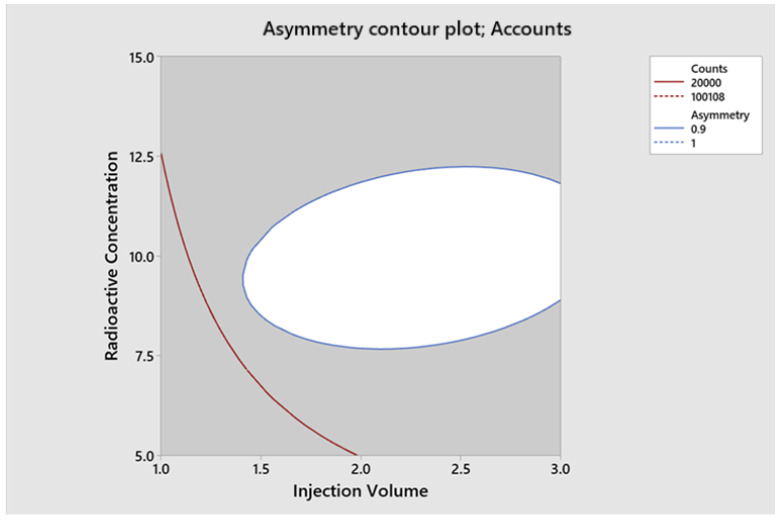
^131^I Graphical Optimization.

The analysis of the response optimization in the operable method design region (MODR) for the response variables counts and asymmetry (Table 5). In Figure 11, as are the optimal values to be configured for the analytical procedure for the determination ofthe radiochemical purity of ^131^I sodium iodide oral solution (Figure 12).

**Table 5:** Prediction of multiple response.

Variable	Setting Value
Sample volume	3
Radioactive concentration	10
Carrier volume	40

**Response****Adjustment****EE of** **Adjustment****95% CI****95% PI**Asymmetry0.90190.0153(0.8627; 0.9412)(0.8420; 0.9619)Counts62,2141931(57,249; 67,178)(54,631; 69,797)

Solution
**Solution****Sample Volume****Radioactive Concentration****Carrier Volume****Asymmetry Adjustment****Counts** **Adjustment****Compound** **Desirability**1310400.90193862,213.80.727118

### 2.6. Selectivity Determination

**Figure 13:**
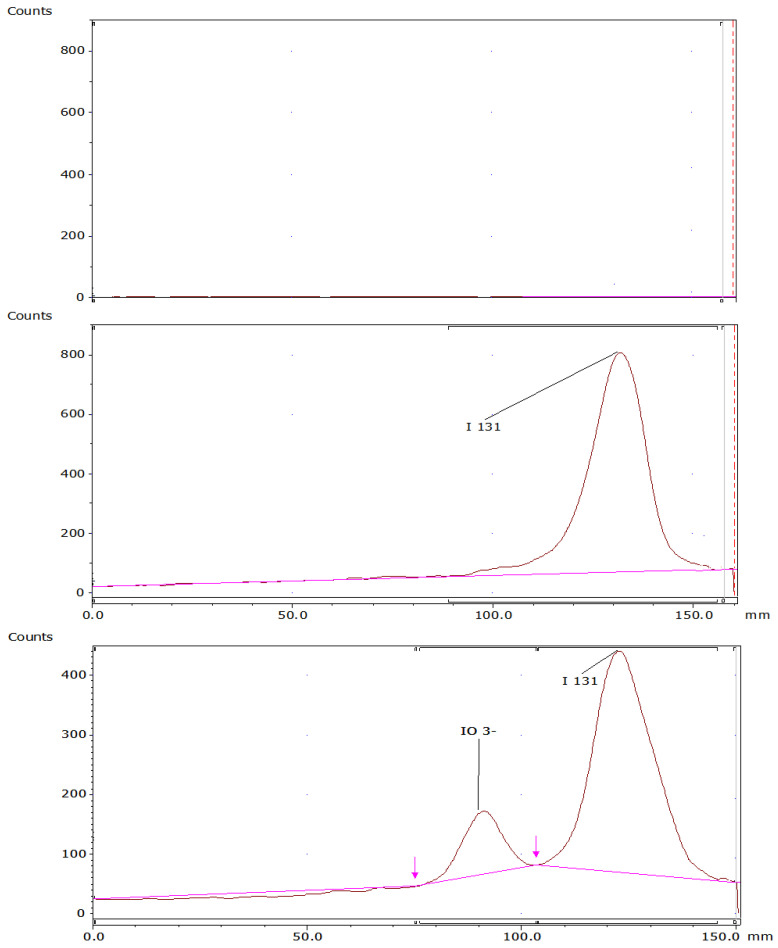
TLC scanner chromatograms: blank, ^131^I sample, radiochemical impurity (reduced): IO_3_^−^.

The selectivity analysis compared the response of blanks and/or placebos and degraded samples to the sample at room temperature. The method was able to distinguish between all possible chemical species that can be generated (Figure 13). The TLC scanner method displayed Retention factor (Rf) values in the chromatograms for the ^131^I iodide band, with an Rf of 0.84, and the radiochemical impurity IO_3_^−^, with an Rf of 0.50 to 0.55, without any chromatographic peaks from the blank and/or placebo, indicating that there was no interference, and the Rf values for the iodide ^131^I band and the radiochemical impurity are distinct.

### 2.7. Linearity Determination

**Figure 14:**
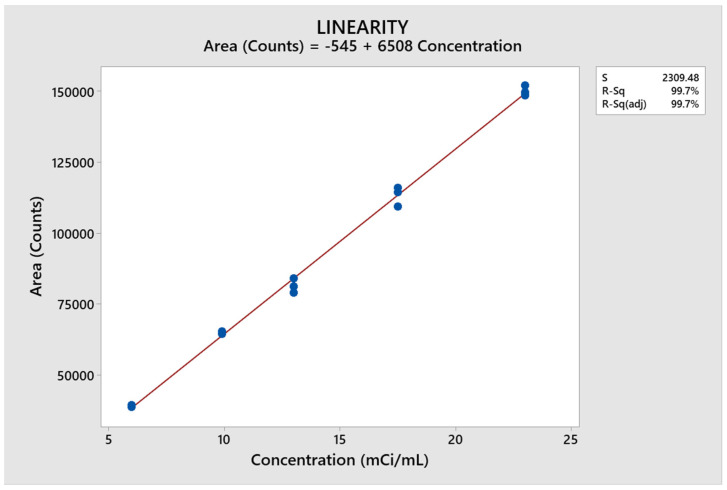
Linearity ^131^I.

The measured values for the ^131^I solutions are shown in Table 6. Here, we can see that there is a clear trend to linearity, because when the radioactive concentration of ^131^I is increased, counts are increased proportionally. Using these data, we constructed the regression curve for the concentration of ^131^I (Figure 14).

The results were statistically analyzed by assessing the radioactive concentrations (mCi/mL) in relation to the corresponding area (Counts). The equation of the line is shown in Figure 14, where:

y = 6,507,675x ± 545,389

The values achieved by regression statistics were a Pearson correlation coefficient (r) of 0.998 and a coefficient of determination (r2) of 0.997. The coefficient of determination indicates that there is good linearity and provides greater statistical significance.

Using the t-student test and ANOVA, the “t” statistic was found, along with a variance test. The regression value of 648.19 demonstrated that the correlation is linear and significant. The *p*-value of 0.000 indicated that the analysis of variance has a high correlation, so it can be inferred that the method is linear.

### 2.8. Determination of Precision

Instrumental repeatability, method repeatability, and intermediate precision were evaluated in the analysis for the parameter precision. It was observed that the RSD of the instrument repeatability area was 0.935%. The RSD of the method repeatability was 1.266%, with an individual confidence interval of 96.84% to 102.66% conformed and an average confidence interval of 99.75 ± 0.971% conformed. For intermediate precision, different equipment and analysts were evaluated. Equipment 1 is the TLC scanner, from the brand Scan-Ram, the model SR1A, and the code PM05AC. Equipment 2 is a single-channel spectrophotometric chain from the brand Canberra. Pass results were obtained, and the overall RSD was 0.624%, as shown in Table 7.

### 2.9. Robustness Determination

**Figure 15:**
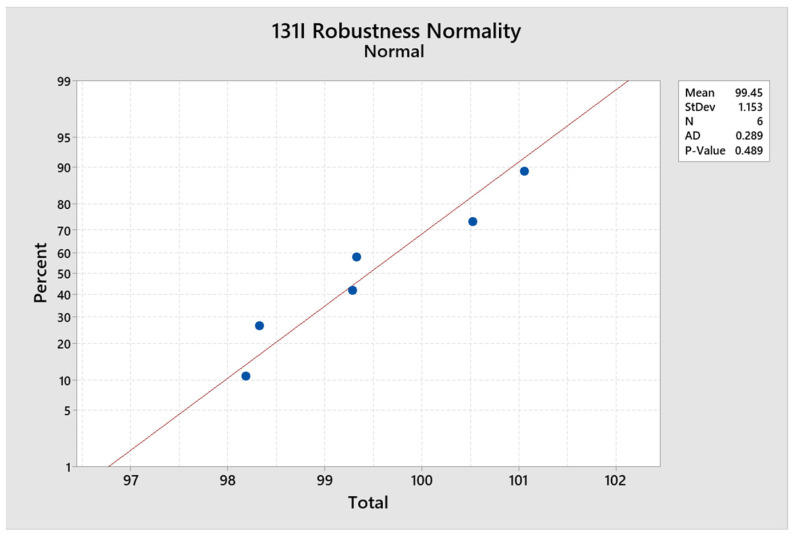
Normal robustness of ^131^I.

For this parameter, the condition of variability was evaluated. For this case, condition 1’s stationary phase was 70% methanol, and for Condition 2, it was 60% methanol. The overall RSD with all results was 0.101%, the overall normal was 0.654 (Figure 15), the normality of the first condition was 0.563, the normality of the second condition was 0.601, and the statistical test t was 0.901; compliant results were obtained.

### 2.10. Determination of the Limit of Detection and Quantitation

The detection limit is the concentration derived from the smallest response that can be detected with reasonable certainty for a given analytical procedure [10]. It was determined by analyzing samples with known concentrations. 

The detection limit was calculated with the formula [10]:

DL = (s × 3.3)/Slope

where s is the standard deviation of the response. 

The limit of quantitation (LOQ) was calculated using the formula [10]:

QL = (s × 10)/Slope

By interpolating the regression equation, the limit of detection value was calculated to obtain a radioactive concentration of 0.09 mCi/mL, and the limit of quantitation value was calculated to obtain a radioactive concentration of 0.53 mCi/mL, obtaining compliant results.

### 2.11. Rank Determination

The working range for the validation parameters was established between 6.00 mCi/mL and 22.0 mCi/mL, by demonstrating precision and linearity over that range for the validated analytical procedure.

### 2.12. Sample Solution Stability Determination

**Figure 16:**
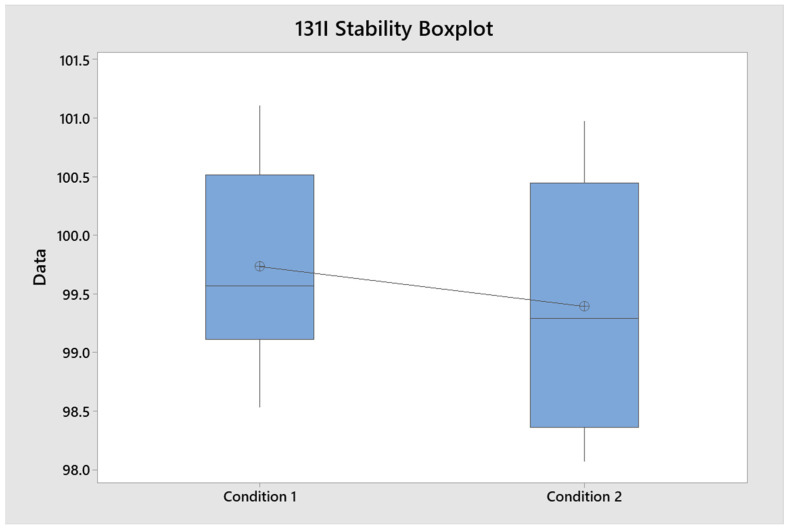
^131^I stability box plot.

The stability parameter of the sample solution was evaluated at 0 h, 2 h, and 20 h time points, and the data distribution was also evaluated. The overall RSD with all 0-, 2-, and 20-h sample results was 0.362%. The normality of the samples at 0 h was 0.206, the normality of the samples at 20 h was 0.733 and the t-statistical test was 0.568, obtaining compliant results, and it was determined that the prepared samples could be processed up to 20 h later (Figure 16).

## 3. Materials and Methods

The analytical quality by design (AQbD) life cycle of the radiochemical purity analytical methodology, using a TLC scanner for the radiopharmaceutical product sodium iodide ^131^I oral solution, applies tools such as the ATP (analytical target profile), determination of analytical procedure parameters, CQA (critical quality attributes), quality risk management, optimization and method development with DoE and MODR (method operable design region), AQbD method validation, routine use, and continuous monitoring. The validation, as it is a non-pharmacopeial methodology, was based on the validation parameters indicated in the USP and the ICH Q2 (R2) guidelines, for the following parameters: selectivity, linearity, precision, robustness, detection limit (DL), limit quantitation (QL), sample stability, and range. The results obtained were statistically analyzed for the development of the AQbD.

### 3.1. Process Description

The implementation of AqbD in the analytical process was developed in the Quality Control Department, Radiochemistry Laboratory I (Laboratory No. 19) of the IPEN Radioisotope Production Plant. The equipment used was a radio–TLC scanner with the code PM05AC from the brand: Scan-RAM, as the model SR1A with the serial number SR1A/0117/380, and a single-channel gamma spectrometry chain with the code PM03AC from the brand Canberra.

### 3.2. Sampling Plan

The samples used were provided by the radiopharmaceutical section of the production department and delivered to the analytical development section of the quality control department.

### 3.3. Analytical Method

The analytical method used was the test of radiochemical purity of sodium iodide ^131^I oral solution.

#### 3.3.1. Equipment, Materials and Reagents

Materials:

- Chromatographic paper No. 1 of 10 mm× 200 mm.
- Chambers or chromatographic tanks.
- Micropipette from 2 to 20 µL (code: TH80AC) and micropipette from 20 to 200 µL (code: TH82AC).

Equipment:

- Radio–TLC scanner (code: PM05AC, brand: Scan-RAM).
- Single-channel gamma spectrometry chain (code: PM03AC, brand: Canberra).
- Dose calibrator (code: CA10AC, brand: Capintec).
- Radiation Monitor (code: DP28JP, brand: Technical Associates).
- ^131^I radiochemical fume hood.
- Handheld dosimeter.
- Body dosimeter.

Reagents:

- Methanol ACS
- Potassium iodide ACS
- ACS sodium bicarbonate
- Starch SR
- Potassium iodate
- Hydrogen peroxide

#### 3.3.2. Chromatographic System for Thin-Layer Chromatography (TLC)

- Detector: NaI (sodium iodide).
- Mobile phase: 70% methanol.
- Stationary phase: Chromatographic Paper No. 1.
- Volume: 3 µL.
- Time: 90 min approximately.

### 3.4. Analytical Quality by Design

The AQbD approach to the analytical procedure for the determination of radiochemical purity by a thin-layer chromatography (TLC) method with a radio–TLC scanner system was based on prior knowledge and internal evaluation, quality risk management, DoE, and MODR.

### 3.5. Implementation of the Analytical Quality by Design (AQbD) Approach

- Analytical target profile (ATP): a prospective summary of performance characteristics was performed [eleven].
- Determination of the parameters of the analytical procedure and CQA (critical quality attributes): critical analytical attributes (CAA) and critical method variables (CMV) were identified.
- Risk management (QRM): an Ishikawa spine diagram, failure mode and effects analysis (FMEA) were used.
- Design space (DoE): a response surface (MSR) methodology of the Box–Behnken design (BBD) was generated.
- Method operable design region (MODR): Optimum fit points and combined ranges are defined for two or more variables, within which the analytical procedure is shown to be fit for its intended use.
- Control strategy: it was established based on the experimental data collected during the CQA, DoE, and MODR stages.

### 3.6. Validation of the Analytical Quality by Design (AQbD) Method

The AQbD method validation approach establishes the validation strategy of the analytical procedure for the determination of radiochemical purity by means of a thin-layer chromatography (TLC) method with a radio–TLC scanner for method operable design region (MODR) and the control strategy.

### 3.7. Parameters

- Selectivity: two sample solutions, two sample solutions with radiochemical impurity IO_3_^−^ reduced, two placebo solutions, and one diluent sample or mobile phase sample were prepared.
- Linearity/response function: five concentration levels and triplicate analysis were considered for each radioactive concentration of 6.0, 10.0, 13.0, 17.0, and 22.0 mCi/mL. A regression curve was made in mCi/mL of the reading of the areas (counts) at different radioactive concentrations.
- Precision: one sample solution of sodium iodide ^131^I oral solution was prepared. Solutions were prepared at concentrations of 6.0, 10.0, and 22.0 mCi/mL. Six tests were carried out with different teams and quality control specialists.
- Robustness: the variability factor contributed by the change of type of mobile phase was evaluated, which was 60% methanol.
- Detection limit (DL) and quantitation limit (QL): analysis was performed in triplicate for each radioactive concentration between 6.00 and 22.0 mCi/mL.
- Range: the parameters of precision, linearity, DL, and QL were evaluated.
- Stability of the sample solution: the stability was evaluated at 0 h up to 20 h after its preparation.

### 3.8. Work Solutions

- Solution A: weigh approximately 100 mg of potassium iodide, 200 mg of potassium iodate, 1 g of sodium bicarbonate to a 100 mL volumetric flask, add 20 mL of distilled water, and dissolve, make up to volume with distilled water, and homogenize.
- Sample solution: the approximate radioactive concentration is 10.0 mCi/mL. Dilute the sodium iodide ^131^I product solution with diluent solution to obtain a concentration of 10.0 mCi/mL.
- Placebo solution: the placebo delivered by the production area was used.
- Blank solution: the diluents used in the procedure were used.
- Mobile phase: transfer approximately 7 mL of methanol ACS to a 10 mL volumetric flask, make up to volume with distilled water, and mix.
- Acidified hydrogen peroxide solution: add 6 drops of 1 N hydrochloric acid to 10 mL of hydrogen peroxide solution.

### 3.9. Procedure

The procedure was performed using nitrile gloves, a metal clamp, a radiochemical hood, a leaded visor, lead shielding, and adequate lighting.

Chromatographic strips No. 1 were placed on a paper towel with the help of a clamp and activated in an oven at 70 °C for 30 min; once activated, remove and use immediately or store for 24 h in a desiccator on activated silica gel.

Then, the activated strip was placed on a towel and placed in the seeding area of the laboratory. A smooth line was marked 1.5 cm from the start, and solution A was placed in the sowing area, 20 mm from the end of the strip, leaving it to dry at room temperature. Three µL of the ^131^I sample solution was seeded with a micropipette at one end of the strip; then, it was dried at room temperature for approximately 1 min.

Subsequently, the strip was placed in the chromatographic tank previously equilibrated in the mobile phase, approximately 1 mm from the surface of the solvent. Care was taken that the solvent came into direct contact with the seeding point and that the edges of the strip did not come into contact with the edge of the tank. The chromatogram was then run until the mobile phase front had traveled 160 mm down the strip, was removed, and was allowed to air dry.

System adequacy: the sample solution was injected in duplicate. The relative standard deviation for both radiochemical purity runs PRQ1 and PRQ2 should not be greater than 2.0%.

Specification: The radioactivity of the iodide ^131^I band is not less than 95% of the total radioactivity and its Rf value is within a range of ±5% of the value found for sodium iodide, determined under parallel conditions.

Radiochemical purity: ≥95% as ^131^I iodide of total radioactivity.

## 4. Conclusions

After the detailed evaluation of the AQbD results for the radiochemical purity method (PQR) by TLC scanner for the radiopharmaceutical sodium iodide ^131^I oral solution, there was a deeper understanding and optimization of the method and its analytical process through the analytical target profile (ATP), the critical quality at-tributes (CQA), quality risk management (QRM), design of experiments (DoE), method operable design region (MODR), method validation, and continuous monitoring. The critical method variables (CMV) and the critical analytical attributes (CAA) were extracted through prior knowledge and internal evaluation, as outlined in Table 2. Quality risk management (QRM) highlighted key process parameters (factors or operational steps of the analytical procedure) such as injection volume, radioactive concentration, and carrier volume. The design of experiments (DoE) confirmed the critical method variables based on statistical significance: for the response variable counts with *p* < 0.05, an R2 value of 99.49%, and an adjR2 of 99.35%, the are injection volume and radioactive concentration; and for asymmetry, with *p* < 0.05, an R2 value of 96.35%, and an adjR2 of 92.70%, they are injection volume and carrier volume. Appropriate optimal ranges for method variables were established. The method operable design region (MODR), based on method factors and settings, establishes the settings for the injection variable as 3 μL, the radioactive concentration as 10 mCi/mL, and the carrier volume as 40 μL. The validation of the AQbD method, using the knowledge of DoE and MODR, statistically demonstrated that the method is selective because it allows for the distinguishing of chemical species that are generated without interference, for both the iodide ^131^I band, with an Rf of 0.8, and the radiochemical impurity IO_3_^−^, with an Rf of 0.6, with a linear response at the five concentration levels from 6.0 to 22.0 mCi/mL with a Pearson correlation coefficient (r) of 0.998 and a coefficient of determination (r^2^) of 0.997, demonstrating the accuracy of the method with a global RSD of 0.624%, the robustness of different stationary phases with a global RSD of 0.101%. It is also capable of detecting small amounts of radioactive concentrations and obtaining a detection limit of 0.09 mCi/mL and a quantification limit of 0.53 mCi/mL, a range of conforming parameters, and stability in processing the samples evaluated at various time points (at 0, 2 and 20 h) with a global RSD of 0.362%.

AQbD parameters such as ATP, CQA, quality risk management, optimization and method development with DoE, MODR, and method validation (including selectivity, linearity, precision, robustness, limit of detection, limit of quantification, range, and solution stability) collectively demonstrate the method’s robustness and its capacity to consistently produce reliable, high-quality data throughout the analytical process [14,15].

## Figures and Tables

**Table 1:** Analytical target profile (ATP).

Analytical Target Profile (ATP) Element	Target/Requirement	Rationale
Analytical profile	Determination of the radiochemical purity of the radiopharmaceutical sodium iodide ^131^I oral solution capable of detecting radiochemical impurities and interferences.	To determine the purity of the radioactive iodide ^131^I in the radiopharmaceutical Sodium iodide ^131^I oral solution.
Instrumentation/method type/detection mode/chromatography	TLC scanner, gamma spectroscopy, NaI scintillation radiation detector (Tl), and thin-layer chromatography.	A gamma ray interacts with a scintillator and produces a light pulse that is converted into an electric pulse by a photomultiplier tube (PMT).
Specificity	Blank, placebo, and no interference from radiochemical impurities should be observed.	The method must be specific and must be able to distinguish radioactive impurities from radioactive iodide ^131^I.
Intermediate precision/instrumental and method repeatability	The overall RSD with all results below 3%.	ICH Q2 (R2) guideline requirements
Linearity	The correlation coefficient and the determination should not be less than 0.99.	Linearity must be obtained at different levels of radioactive concentration. ICH Q2 (R2) guideline requirements.
Robustness	The overall RSD with all results below 2%.	The test results should not be affected by small changes in the method parameters.
Sample stability	The overall RSD with all results below 3%.	Test results should not be affected by preparation time when processed.
Detection limit/quantitation limit	Minimum quantity that can be detected and can be determined precisely.	ICH Q2 (R2) guideline requirements.

**Table 2:** Critical quality attributes (CQAs).

Critical Method Variables (CMV)	Critical Analytical Attributes (CAA)
Injection volume	Counts
Sample concentration	Delay factor (RF)
Carrier	Asymmetry (T)
Mobile phase	
Stationary phase	
Counting speed	
Development time	
Detection	

**Table 3:** Risk estimate matrix.

CAA	Detector	Chromatography Plate	Carrier	Mobile Phase	Injection Volume	Radioactive Concentration
Counts	High	Low	Medium	Low	High	High
Delay Factor	Low	Medium	High	High	Low	Low
Asymmetry	Low	Medium	High	Low	Medium	Low

**Table 6:** Total counts of ^131^I.

Levels (mCi/mL)	Area (Counts) Average
6.00	39,143.333 ± 0.7825
10.00	65,067.333 ± 0.6271
13.00	81,480.666 ± 3.0296
17.50	113,205.33 ± 2.9852
23.00	150,009.00 ± 1.1630

**Table 7:** Intermediate precision of ^131^I.

Analyst Equipment	Area Counts	PRQ (%)	RSD (%)	%RSD Overall
Analyst 1	64,930	101.05	1.1375	0.624
Equipment 1	65,526	98.185		
	66,521	99.327		
	63,468	98.392		
	65,784	98.015		
	63,891	99.102		
Analyst 2	11,235	99.902	2.6032	
Equipment 2	11,766	99.958		
	11,845	99.890		
	11,716	99.923		
	12,126	99.844		
	11,992	99.825		

## Data Availability

Data are contained within the article.
